# A Provably-Secure ECC-Based Authentication Scheme for Wireless Sensor Networks

**DOI:** 10.3390/s141121023

**Published:** 2014-11-06

**Authors:** Junghyun Nam, Moonseong Kim, Juryon Paik, Youngsook Lee, Dongho Won

**Affiliations:** 1 Department of Computer Engineering, Konkuk University, 268 Chungwondaero, Chungju, Chungcheongbukdo 380-701, Korea; E-Mail: jhnam@kku.ac.kr; 2 Information Management Division, Korean Intellectual Property Office, 189 Cheongsaro, Daejeon 302-701, Korea; E-Mail: moonseong@kipo.go.kr; 3 Department of Computer Engineering, Sungkyunkwan University, 2066 Seoburo, Suwon, Gyeonggido 440-746, Korea; E-Mail: wise96@skku.edu; 4 Department of Cyber Investigation Police, Howon University, 64 3-gil, Gunsan, Jeonrabukdo 573-718, Korea; E-Mail: ysooklee@howon.ac.kr

**Keywords:** wireless sensor network, authentication scheme, authenticated key exchange, user anonymity, smart card, two-factor security

## Abstract

A smart-card-based user authentication scheme for wireless sensor networks (in short, a SUA-WSN scheme) is designed to restrict access to the sensor data only to users who are in possession of both a smart card and the corresponding password. While a significant number of SUA-WSN schemes have been suggested in recent years, their intended security properties lack formal definitions and proofs in a widely-accepted model. One consequence is that SUA-WSN schemes insecure against various attacks have proliferated. In this paper, we devise a security model for the analysis of SUA-WSN schemes by extending the widely-accepted model of Bellare, Pointcheval and Rogaway (2000). Our model provides formal definitions of authenticated key exchange and user anonymity while capturing side-channel attacks, as well as other common attacks. We also propose a new SUA-WSN scheme based on elliptic curve cryptography (ECC), and prove its security properties in our extended model. To the best of our knowledge, our proposed scheme is the first SUA-WSN scheme that provably achieves both authenticated key exchange and user anonymity. Our scheme is also computationally competitive with other ECC-based (non-provably secure) schemes.

## 1. Introduction

As various sensors emerge and the related technologies advance, there has been a dramatic increase in the interest in wireless sensor networks (WSNs). Today, billions of physical, chemical and biological sensors are being deployed into various types of WSNs for numerous applications, including military surveillance, wildlife monitoring, vehicular tracking and healthcare diagnostics [1]. A major benefit of WSN systems is that they provide unprecedented abilities to explore and understand large-scale, real-world data and phenomena at a fine-grained level of temporal and spatial resolution. However, providing an application service in a WSN environment introduces significant security challenges to be addressed among the involved parties: users, sensors and gateways. One important challenge is to achieve authenticated key exchange between users and sensors (via the assistance of a gateway), thereby preventing illegal access to the sensor data and their transmissions. Authenticated key exchange in WSNs is more challenging to achieve than in traditional networks due to the sensor network characteristics, such as resource constraints, unreliable communication channel and unattended operation. Another important challenge is to provide user anonymity, which makes authenticated key exchange even harder. As privacy concern increases, user anonymity has become a major security property in WSN applications, as well as in many other applications, like mobile roaming services, anonymous web browsing, location-based services and e-voting. User authentication schemes for WSNs are designed to address these security challenges [2,3], and are a subject of active research in network security and cryptography.

Generally speaking, the design of cryptographic schemes (including user authentication schemes for WSNs) is error-prone, and their security analysis is time-consuming. The difficulty of getting a high level of assurance in the security of cryptographic schemes is well illustrated with examples of flaws discovered in many such schemes years after they were published; see, e.g., [4–6]. The many flaws identified in published schemes over the decades have promoted formal security analyses, which are broadly classified into two approaches [7,8]: the computer security approach and the computational complexity approach. The computer security approach places its emphasis on automated machine specification and analysis mostly in the Dolev–Yao adversarial model [9], where the underlying cryptographic primitives are often used in a black-box manner ignoring some of cryptographic details. The main problem with this automated approach is intractability and undecidability, as the adversary may exhibit a large set of possible behaviors, which leads to a state explosion. Cryptographic schemes proven secure in such a fashion could possibly be flawed, yielding a false positive result. In contrast, the computational complexity approach places its emphasis on deriving a polynomial-time reduction from the problem of breaking the scheme into another problem believed to be hard. A complete computational proof under a well-established cryptographic assumption provides a strong assurance that the security properties of the scheme are satisfied. Accordingly, it has been standard practice for the designers of cryptographic schemes to provide a proven reduction for the security of their schemes in a widely-accepted model [10,11]. Although these human-generated mathematical proofs are usually lengthy and complicated, they are certainly an invaluable tool for getting secure cryptographic schemes.

In 2009, Das [12] proposed a smart-card-based user authentication scheme for wireless sensor networks; throughout the paper, we call such a scheme a SUA-WSN scheme. Since then, the design of SUA-WSN schemes has received significant attention from researchers due to their potential to be widely deployed, and a number of solutions offering various levels of efficiency and security have been subsequently proposed [2,3,13–27]. Early schemes only aimed to achieve mutual authentication [13–15], while later schemes attempted to provide additional security properties, such as authenticated key exchange [2,3,16–27] and user anonymity [2,3,20,22–24,26]. Some schemes [16,21,27] employ elliptic curve cryptography to provide perfect forward secrecy, while others [2,3,12–14,17–20,22–26] only use symmetric cryptography and hash functions to focus on improving the efficiency.

One important security requirement for SUA-WSN schemes is to ensure that only a user who is in possession of both a smart card and the corresponding password can pass the authentication check of the gateway and gain access to the sensor network and data. A SUA-WSN scheme that meets this requirement is said to achieve two-factor security. To properly capture the notion of two-factor security, the adversary against SUA-WSN schemes is assumed to be able to either extract the sensitive information in the smart card of a user possibly via a side-channel attack [28,29] or learn the password of the user through shoulder-surfing or by exploiting a malicious card reader, but not both. Clearly, there is no means to prevent the adversary from impersonating a user if both the password of the user and the information in the smart card are disclosed.

Despite the research efforts over the recent years, it remains a significant challenge to design a robust SUA-WSN scheme that carries a formal proof of security in a widely-accepted model. As summarized in Table 1, most of the published schemes either provide no formal analysis of security [3,12–14,16,20–22,24–26] or fail to achieve important security properties, such as mutual authentication, session-key security, user anonymity, two-factor security and resistance against various attacks [3,13–16,19,21–27,30,31]. Some schemes [2,17–19,23,27] have been proven secure using a computer security approach, which, as mentioned above, suffers from intractability and undecidability and could possibly give a false positive result. To the best of our knowledge, Chen and Shih's scheme [15] is the only SUA-WSN scheme that was proven secure using a computational complexity approach. However, Chen and Shih's scheme does not provide key exchange functionality, but only focuses on mutual authentication (and thus, inherently, cannot carry a proof of authenticated key exchange). Moreover, the security model used for this scheme captures neither the user anonymity property nor the notion of two-factor security.

The contributions of this paper are two-fold:

(1) We present a security model for the analysis of sua-wsn schemes. Our security model is derived by extending the widely-accepted model of Bellare, Pointcheval and Rogaway [10] to incorporate into it the user anonymity property and the notion of two-factor security. Notice that the original Bellare–Pointcheval–Rogaway (BPR) model for authenticated key exchange (AKE) already captures insider attacks, offline dictionary attacks and other common attacks. We refer readers to [32] to understand how a key exchange scheme that is vulnerable to an offline dictionary attack can be rendered insecure in the BPR model. Our extension of the BPR model provides two security definitions, one for the AKE security and one for the user anonymity property, and both definitions capture the notion of two-factor security. Security properties like authentication, session-key security, perfect forward secrecy, known-key security and resistance against insider attacks and offline dictionary attacks are implied by the AKE security.
(2) We propose the first SUA-WSN scheme whose AKE security, as well as user anonymity are formally proven in a widely-accepted model. Our scheme employs elliptic curve cryptography (ECC) to provide perfect forward secrecy, but differs from other ECC-based schemes [16,21,27] in that it provides user anonymity. We prove the security properties of our scheme in the random oracle model under the elliptic curve computational Diffie–Hellman (ECCDH) assumption. We also show that our provably-secure scheme is computationally competitive compared with other ECC-based (non-provably secure) schemes.

Table 2 shows the basic notation that is used consistently throughout this paper.

The remainder of this paper is structured as follows. Section 2 describes our extended security model for the analysis of SUA-WSN schemes. Section 3 presents the proposed SUA-WSN scheme along with cryptographic primitives on which the security of the scheme relies and then compares our scheme with other ECC-based schemes, both in terms of efficiency and security. Section 4 provides proofs of the user anonymity property and the AKE security for our scheme. Section 5 concludes the paper, summarizing our result and presenting some interesting future work.

## 2. Our Extended Security Model for SUA-WSN Schemes

In this section, we present a security model extended from the BPR model [10] to capture the security properties of SUA-WSN schemes.

*Participants and long-lived keys*. Let *GW* be the gateway and 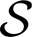
*ℛ* 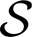 and 
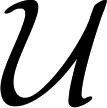
*ℛ* 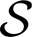 be the sets of all sensors and users, respectively, registered with *GW*. Let *ɛ* = {*GW*} ∪ 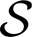
*ℛ* 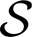 ∪ 
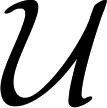
*ℛ* 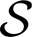. We identify each entity *E* ∈ *ɛ* by a string, and interchangeably use *E* and *ID_E_* to refer to this identifier string. To properly capture the user anonymity property, we assume that: (1) each user *UR* ∈ 
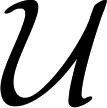
ℛ 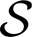 has its pseudo identity *PID_UR_* (as well as its true identity *ID_UR_*); and (2) the adversary 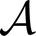 is given only *PID_UR_*, but not *ID_UR_*. A user *UR* may run multiple sessions of the authentication and key exchange protocol of the scheme (hereafter simply called the protocol), either serially or concurrently, to anonymously establish a session key with a sensor *SR* ∈ 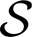
ℛ 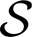 via the assistance of the gateway *GW*. Therefore, at any given time, there could be multiple instances of the entities *UR, SR* and *GW*. We use $\Pi_{E}^{i}$ to denote instance *i* of entity *E* ∈ *E*. Instances of *UR* and *SR* are said to accept when they compute a session key in an execution of the protocol. We denote the session key of $\Pi_{E}^{i}$ by $sk_{E}^{i}$. Before the protocol is ever executed,

- *GW* generates its master secret(s), issues a smart card to each *UR* ∈ 
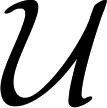
ℛ 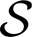 and establishes a shared key with each *SR* ∈ 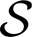
ℛ 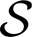; and
- each *UR* ∈ 
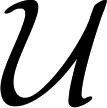
ℛ 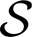 chooses its private password *pw_UR_* from the set of all possible passwords.

*Partnering*. Informally, we say that two instances are partners (or partnered) if they participate together in a protocol session and establish a shared key. Formally, the partner relationship between instances is defined in terms of the notion of the session identifier. A session identifier (*sid*) is literally an identifier of a protocol session and is typically defined as a function of the messages exchanged in the session. Let $sid_{E}^{i}$ denote the *sid* of instance $\Pi_{E}^{i}$. We say that two instances, $\Pi_{UR}^{i}$ and $\Pi_{SR}^{j}$, are partners if: (1) both instances have accepted; and (2) $sid_{UR}^{i}=sid_{SR}^{j}$.

*Adversary capabilities*. The adversary 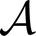 is a probabilistic polynomial-time (ppt) machine, which has full control of all communications between entities. More specifically, the PPT adversary 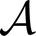 is able to: (1) eavesdrop, modify, intercept, delay and delete the protocol messages; (2) ask entities to open up access to session keys and long-term keys; and (3) extract the sensitive information on the smart cards of users. These capabilities of 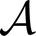 are modeled using a pre-defined set of oracles to which 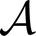 is allowed to ask queries. We assume that, when making oracle queries directed at (instances of) *UR*, the adversary 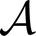 uses the pseudo identity *PI D_UR_*, since it does not know the true identity *ID_UR_*. The oracle queries are described as follows:

- Execute ( $\Pi_{UR}^{i}$, $\Pi_{SR}^{j}$, $\Pi_{GW}^{k}$): This query models passive eavesdropping on the protocol messages. It prompts a protocol execution among the instances $\Pi_{UR}^{i}$, $\Pi_{SR}^{j}$ and $\Pi_{GW}^{k}$ and returns the transcript of the protocol execution to 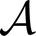.
- $\text{Send}(\prod_{E}^{i},m)$, *m*): This query sends a message *m* to an instance $\Pi_{E}^{i}$, modeling active attacks against the protocol. Upon receiving *m*, the instance $\Pi_{E}^{i}$ proceeds according to the protocol specification. Any message generated by $\Pi_{E}^{i}$ is output and given to 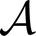. A query of the form Send( $\Pi_{UR}^{i}$, start) prompts $\Pi_{UR}^{i}$ to initiate a protocol session.
- Reveal( $\Pi_{E}^{i}$): This query captures known key attacks. Upon receiving this query, the instance $\Pi_{E}^{i}$ returns its session key $sk_{E}^{i}$ back to 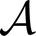 (if it has accepted).
- CorruptLL(*E*): This query returns the long-lived secret(s) of entity *E*, capturing the notion of forward secrecy, as well as resistance to unknown key share attacks and insider attacks.
- CorruptSC(*UR*): This query captures side-channel attacks (*i.e.*, the notion of two-factor security) and returns the information stored in the smart card of *UR*.
- TestAKE( $\Pi_{E}^{i}$): This query is used for defining the indistinguishability-based security of session keys. The output of the query depends on a random bit *b* chosen by the oracle; in response to the query, either the real session key $sk_{E}^{i}$ if *b* = 1 or a random key drawn from the session-key space if *b* = 0 is returned to 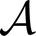.
- TestID(*UR*): This query is used for determining whether the protocol provides user anonymity or not. Depending on a random bit *b* chosen by the oracle, 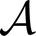 is given either the identity actually used for *UR* in the protocol sessions (when *b* = 1) or a random identity drawn from the identity space (when *b* = 0).

*SR* and *GW* are said to be corrupted when they are asked a CorruptLL query, while *UR* is considered as corrupted if it has been asked both CorruptLL and CorruptSC queries.

*Authenticated key exchange (AKE)*. We define the AKE security of the authentication and key exchange protocol *P* by using the notion of freshness of instances. Informally, a fresh instance refers to an instance whose session key should be kept indistinguishable from a random key to the adversary 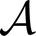, and an unfresh instance refers to an instance that holds a session key that can be distinguishable from a random key by trivial means. A formal definition of freshness follows:

**Definition 1** (Freshness). *An instance* $\Pi_{E}^{i}$ *is fresh unless one of the following occurs*:

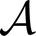
 *queries* Reveal( $\Pi_{E}^{i}$) *or* Reveal( $\Pi_{E}^{j}$), where $\Pi_{E}^{j}$, is the partner of $\Pi_{E}^{i}$;

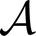
 *queries* CorruptLL(*SR*) *or* CorruptLL(*GW*) *before* $\Pi_{E}^{i}$ *accepts*.

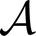
 *queries both* CorruptLL(*UR*) *and* CorruptSC(*UR*), *for some UR* ∈ 
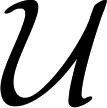
ℛ 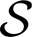, *before* $\Pi_{E}^{i}$ *accepts*.

The AKE security of the protocol *P* is defined in the context of the following two-phase experiment: Experiment **ExpAKE**_0_:

Phase 1. 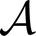 freely asks any oracle queries, except that:

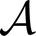
 is not allowed to ask queries of the TestID oracle.

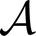
 is not allowed to ask the TestAKE( $\Pi_{E}^{i}$) query if the instance $\Pi_{E}^{i}$ is not fresh.

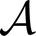
 is not allowed to ask the Reveal( $\Pi_{E}^{i}$) query if it has already asked a TestAKE query of $\Pi_{E}^{i}$ or its partner instance.

Phase 2. When Phase 1 is over, 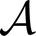 outputs a bit *b′* as a guess of the random bit *b* selected by the TestAKE oracle. 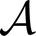 succeeds if *b* = *b′*.

Let SuccAKE_0_ be the event that 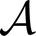 succeeds in the experiment **ExpAKE**_0_. Let ${\text{Adv}}_{P}^{\text{AKE}}(A)$ denote the advantage of 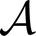 in breaking the AKE security of protocol *P* and be defined as ${\text{Adv}}_{P}^{\text{AKE}}(A)=2$ · Pr*_P_*,_
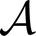
_[SuccAKE_0_] − 1.

**Definition 2** (AKE security). *The authentication and key exchange protocol P is AKE-secure if* ${\text{Adv}}_{P}^{\text{AKE}}(A)$ *is negligible for any PPT adversary* 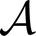.

*User anonymity*. The AKE security does not imply user anonymity. In other words, an authentication and key exchange protocol that does not provide user anonymity may still be rendered AKE secure.

Hence, a new, separate definition is necessary to capture the user anonymity property. Our definition of user anonymity is based on the notion of cleanness.

**Definition 3** (Cleanness). *A user U R* ∈ 
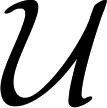
ℛ 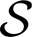 *is clean unless one of the following occurs*:

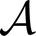
 *queries CorruptLL*(*GW*).

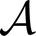
 *queries both CorruptLL*(*UR*) *and CorruptSC(UR)*.

Note that this definition of cleanness does not impose any restriction on asking a CorruptLL query to *SR*. This reflects our objective to achieve user anonymity even against the sensor *SR*.

Now, consider the following experiment to formalize the user anonymity property:

Experiment **ExpID**_0_:

Phase 1. *A* freely asks any oracle queries, except that:

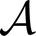
 is not allowed to ask queries of the TestAKE oracle.

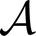
 is not allowed to ask the TestID(*UR*) query if the user *UR* is not clean.

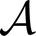
 is not allowed to ask CorruptLL and CorruptSC queries against *GW* and *UR* if it has already asked the TestID(*UR*) query.
Phase 2. When Phase 1 is over, 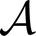 outputs a bit *b′* as a guess on the random bit *b* selected by the TestID oracle. 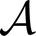 succeeds if *b* = *b′*.

Let SuccID_0_ be the event that *A* succeeds in the experiment **ExpID**_0_. Then, we define the advantage of 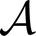 in attacking the user anonymity of protocol *P* as ${\text{Adv}}_{P}^{ID}(A)=2\cdot {\mathrm{Pr}}_{P,A}[{\text{SuccID}}_{0}]-1$.

**Definition 4** (User anonymity). *The authentication and key exchange protocol P provides user anonymity if* ${\text{Adv}}_{P}^{ID}(A)$ *is negligible for any PPT adversary* 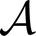.

## 3. The Proposed SUA-WSN Scheme

This section presents our ECC-based user authentication scheme for wireless sensor networks. Our scheme consists of three phases: the registration phase, the authentication, the key exchange phase and the password update phase. We begin by describing the cryptographic primitives on which the security of our scheme relies.

### 3.1. Preliminaries

*Elliptic curve computational Diffie-Hellman (ECCDH) problem*. Let 

 be an elliptic curve group of prime order *q*. Typically, 

 will be a subgroup of the group of points on an elliptic curve over a finite field. Let *P* be a generator of 

. Informally stated, the ECCDH problem for 

 is to compute *xyP* ∈ 

 when given two elements *xP, yP* ∈ 

^2^, where *x* and *y* are chosen at random from ${\mathbb{Z}}_{q}^{*}$. We say that the ECCDH assumption holds in 

 if it is computationally intractable to solve the ECCDH problem for 

. More formally, we define the advantage of an algorithm 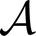 in solving the ECCDH problem for 

 as ${\text{Adv}}_{\text{G}}^{\text{ECCDH}}(A)=\mathrm{Pr}[A(\text{G},xP,yP)=xyP]$. We say that the ECCDH assumption holds in 

 if ${\text{Adv}}_{\text{G}}^{\text{ECCDH}}(A)$ is negligible for all PPT algorithms 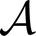. We use ${\text{Adv}}_{G}^{\text{ECCDH}}(t)$ to denote the maximum value of ${\text{Adv}}_{G}^{\text{ECCDH}}(A)$ over all algorithms 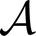 running in time at most *t*.

*Symmetric encryption schemes*. A symmetric encryption scheme Γ algorithms (Enc, Dec) where: (1) the encryption algorithm Enc takes as input an ℓ-bit key *k* and a plain text message *m* and outputs a ciphertext *c*; and (2) the decryption algorithm Dec takes as input a key *k* and a ciphertext c and outputs a message *m*. We require that Dec_*k*_(Enc_*k*_(*m*)) = *m* holds for all *k* ∈ {0,*1*}^ℓ^ and all *m* ∈ 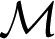, where 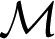 is the plain text space. For an eavesdropping adversary 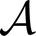 against Γ and for an integer *n* ≥ 1 and a random bit *b* ∈*R* {0,1}, consider the following indistinguishability experiment:
Experiment 
${\text{Exp}}_{\Gamma }^{\text{IND}-\text{EAV}}(A,n,b)$ **for**
*i* = 1 **to**
*n*  *k_i_* ∈*_R_* {0,*1*}^ℓ^   (*m*_0,i_,*m*_1_,*_i_*) ← 

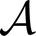
(Γ)  *c*i ← Enc*_ki_*(*m_b,i_*)   

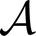
(*c_i_*) *b′* ← 

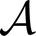
, where *b′* ∈ {0,1} **return**
*b′*

Let ${\text{Adv}}_{\Gamma }^{\text{IND}-\text{EAV}}(A)$ be the advantage of an eavesdropper 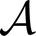 in violating the indistinguishability of Γ, and let it be defined as:

$${\text{Adv}}_{\Gamma }^{\text{IND}-\text{EAV}}(A)=\left|\mathrm{Pr}\left[{\text{Exp}}_{\Gamma }^{\text{IND}-\text{EAV}}\left(A,n,0\right)=1\right]-\mathrm{Pr}\left[{\text{Exp}}_{\Gamma }^{\text{IND}-\text{EAV}}\left(A,n,0\right)=1\right]\right|.$$

We say that Γ is secure if ${\text{Adv}}_{\Gamma }^{\text{IND}-\text{EAV}}(A)$ is negligible in ℓ for any PPT adversary 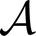. We define ${\text{Adv}}_{\Gamma }^{\text{IND}-\text{EAV}}(t)$ as ${\text{Adv}}_{\Gamma }^{\text{IND}-\text{EAV}}(t)=\max_{A}\left\{{\text{Adv}}_{\Gamma }^{\text{IND}-\text{EAV}}(A)\right\}$, where the maximum is over all PPT adversaries 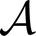 running in time at most *t*.

*Message authentication codes*. A message authentication code (MAC) scheme Δ is a pair of efficient algorithms (Mac, Ver) where: (1) the MAC generation algorithm Mac takes as input an ℓ-bit key *k* and a message *m* and outputs a MAC *δ* and (2) the MAC verification algorithm Ver takes as input a key *k*, a message *m* and a MAC *δ* and outputs one if *δ* is valid for *m* under *k* or outputs zero if *δ* is invalid. Let ${\text{Adv}}_{\Delta }^{EU-\text{CMA}}(A)$ be the advantage of an adversary 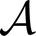 in violating the strong existential unforgeability of Δ under an adaptive chosen message attack. More precisely, ${\text{Adv}}_{\Delta }^{EU-\text{CMA}}(A)$ is the probability that an adversary 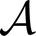, who mounts an adaptive chosen message attack against Δ with oracle access to Mac_*k*_(·) and Ver_*k*_(·), outputs a message/MAC pair (*m,δ*), such that: (1) Verk(*m, δ*) = 1; and (2) *δ* was not previously output by the oracle Mack_*k*_ as a MAC on the message *m*. We say that the MAC scheme Δ is secure if ${\text{Adv}}_{\Delta }^{EU-\text{CMA}}(A)$ is negligible for every PPT adversary 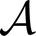. Let ${\text{Adv}}_{\Delta }^{EU-\text{CMA}}(t)$ denote the maximum value of ${\text{Adv}}_{\Delta }^{EU-\text{CMA}}(A)$ over all adversaries 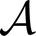 running in time at most *t*.

*Cryptographic hash functions*. Our scheme uses three cryptographic hash functions *L* : {0,1}* → {0,*1*}^ℓ^, H : {0,1}* → {0,1}*^κ^* and *F* : {0,1}* → {0,1}*^ɛ^*, where ℓ is as defined for Δ and Γ, κis the bit-length of session keys and ε is the bit-length of *SID_UR_* (see Section 3.2.1 for the definition of *SID_UR_*). These hash functions are modeled as random oracles in our security proofs.

### 3.2. Description of the Scheme

The public system parameters for our scheme include:

1. an elliptic curve group 

 with a generator *P* of prime order *q*,
2. a symmetric encryption scheme Γ = (Enc, Dec),
3. a MAC scheme Δ = (Mac, Ver), and
4. three hash functions *L, H* and *F*.

We assume that these public system parameters are fixed during an initialization phase and are known to all parties in the network. As part of the initialization, the gateway *GW* chooses two master keys $x,y\in {\mathbb{Z}}_{q}^{*}$, computes its public key *X* = *xP* and establishes a shared secret key *k_GS_* = *L*(*ID_SR_*‖*y*) with each sensor *SR*.

#### 3.2.1. Registration Phase

A user *UR* registers itself with the gateway *GW* as follows:

1. *UR* chooses its identity *I D_UR_* and password *pw_UR_* freely and submits the identity *I D_UR_* to *GW* via a secure channel.
2. *GW* computes *SID_UR_* = Enc*_L_*_(*x*)_(*ID_UR_*‖*ID_GW_*) and issues *UR* a smart card loaded with {*SID_UR_, X, ID_GW_*, 

, *P*, Γ, Δ, *L, H, F*}. (We assume that *q* is implicit in 

.)
3. *UR* replaces *SID_UR_* with *TID_UR_* = *SID_UR_* ⊕ *F*(*ID_UR_*‖*pw_UR_*).

**Figure 1.**
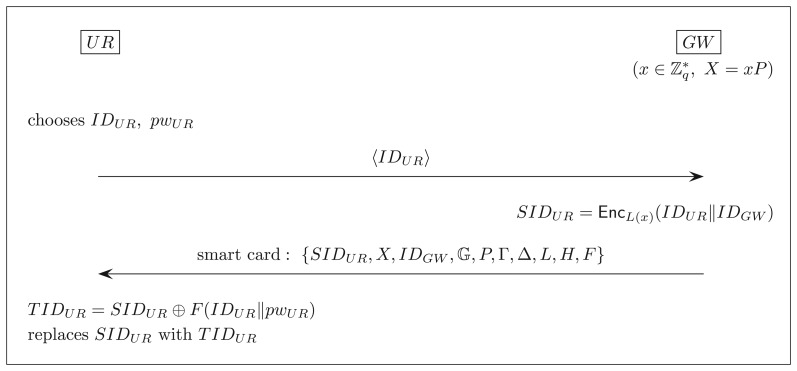
User registration.

This phase of user registration is depicted in Figure 1.

#### 3.2.2. Authentication and Key Exchange Phase

**Figure 2.**
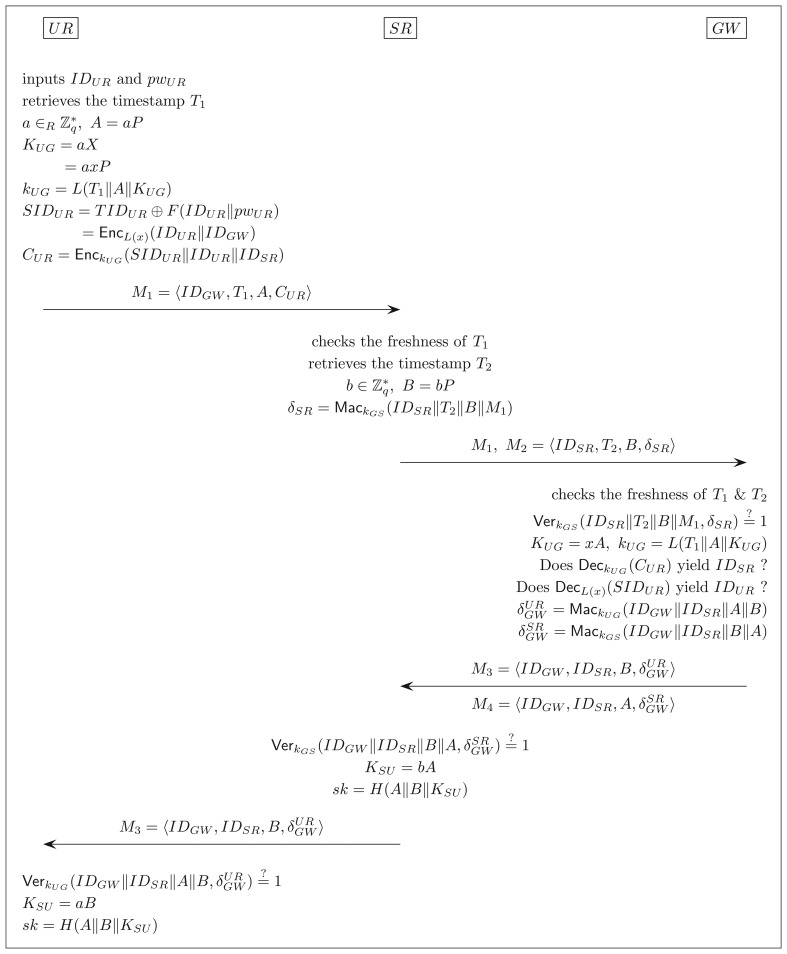
The authentication and key exchange protocol.

*UR* needs to perform this phase with *SR* and *GW* whenever it wishes to gain access to the sensor network and data. The steps of the phase are depicted in Figure 2 and are described as follows:

Step 1. *UR* inserts its smart card into a card reader and inputs its identity *ID_UR_* and password *pw_UR_*. Given *ID_UR_* and *pw_UR_*, the smart card retrieves the current timestamp *T_1_*, selects a random $a\in {\mathbb{Z}}_{q}^{*}$ and computes:
  $$\begin{array}{cc} A & =aP,\\ K_{UG} & =aX\\ & =axP,\\ k_{UG} & =L(T_{1}∥A∥K_{UG}),\\ SID_{UR} & =TID_{UR}⊕F(ID_{UR}∥pw_{UR})\\ & =Enc_{L(x)}(ID_{UR}∥ID_{GW}),\\ C_{UR} & =Enc_{k_{UG}}(SID_{UR}∥ID_{UR}∥ID_{SR}). \end{array}$$
  After the computations, the smart card sends the message *M*_1_ = 〈*ID_GW_, T*_1_, *A, C_UR_*〉 to the sensor *SR*.
Step 2. Upon receiving *M*_1_, *SR* first checks the freshness of *T*_1_. If *T*_1_ is not fresh, *SR* aborts the protocol. Otherwise, *SR* retrieves the current timestamp *T*_2_, chooses a random $b\in {\mathbb{Z}}_{q}^{*}$ and computes *B* and *δ_sr_* as follows:
  $$\begin{array}{cc} B & =bP,\\ \delta_{SR} & ={\text{Mac}}_{k_{GS}}(ID_{SR}∥T_{2}∥B∥M_{1}). \end{array}$$
  Then, *SR* sends the message *M*_2_ = 〈*ID_SR_, T*_2_, *B, δ_SR_*〉 along with *M*_1_ to *GW*.
Step 3 After having received *M*_1_ and *M*_2_, *GW* verifies that: (1) *T*_1_ and *T*_2_ are fresh; and (2) Ver*_kGS_*(*ID_SR_*‖*T*_2_‖*B*‖*M*_1_, *δ_SR_*) = 1. If any of the verifications fails, *GW* aborts the protocol. Otherwise, *GW* computes *K_UG_* = *xA* and *k_UG_* = *L*(*T*_1_‖A‖*K_UG_*), decrypts *C_UR_* with key *k_UG_* and checks if the decryption produces the same *ID_SR_* as contained in *M*_2_. *GW* aborts if the check fails. Otherwise, *GW* decrypts *SID_UR_* with key *L*(*x*) and checks if this decryption yields the same *ID_UR_* as produced through the decryption of *C_UR_*. If only the two IDs match, *GW* computes:
  $$\begin{array}{c} \delta_{GW}^{UR}=Mac_{k_{UG}}\left(ID_{GW}\left\|ID_{SR}\|A\right\|B\right),\\ \delta_{GW}^{SR}=Mac_{k_{GS}}\left(ID_{GW}\left\|ID_{SR}\|B\right\|A\right), \end{array}$$
  and sends two messages *M*_3_ = 〈*ID_GW_, ID_SR_, B*, $\delta_{GW}^{UR}$ 〉 and *M*_4_ = 〈*ID_GW_,ID_sr_,A*, $\delta_{GW}^{SR}$ 〉 to *SR*.
Step 4. When receiving *M*_3_ and *M*_4_, SR verifies that Ver_k_*_GS_*(*ID_GW_*‖*ID_SR_*‖*B*‖*A*, $\delta_{GW}^{SR}$) = 1. If the verification fails, *SR* aborts the protocol. Otherwise, *SR* forwards the message *M*_3_ to *UR* and computes the shared secret *K_SU_* = *bA* and the session key *sk* = *H*(*A*‖*B*‖*K_SU_*).
Step 5. Upon receiving *M*_3_, *UR* checks if *Ver_kUG_*(*ID_GW_*‖*ID_SR_*‖*A*‖*B*, $\delta_{GW}^{SR}$) = 1. *U R* aborts the protocol if the check fails. Otherwise, *U R* computes *K_SU_* = *aB* and *sk* = *H*(*A*‖*B*‖*K_SU_*).

Since *K_SU_* = *bA* = *aB* = *abP, UR* and *SR* will compute the same session key *sk* = H(*A*‖*B*‖*abP*; in the presence of a passive adversary.

#### 3.2.3. Password Update Phase

One of the general guidelines to get better password security is to ensure that passwords are changed at regular intervals. Our scheme allows users to update their passwords at will.

1. *UR* inserts his smart card into a card reader and enters the identity *ID_UR_*, the current password *pw_UR_* and the new password $pw_{UR}^{\prime }$.
2. The smart card computes $TID_{UR}^{\prime }=TID_{UR}⊕F(ID_{UR}∥pw_{UR})⊕F(ID_{UR}∥pw_{UR}^{\prime })$ and replaces *TID_UR_* with $TID_{UR}^{\prime }$.

### 3.3. Performance and Security Comparison

Table 3 compares our scheme with other ECC-based SUA-WSN schemes in terms of the computational requirements, the AKE security and user anonymity. For fairness of comparison, SUA-WSN schemes that use only lightweight symmetric cryptographic primitives are not considered in the table since they cannot achieve forward secrecy, but have a clear efficiency advantage over the ECC-based schemes.

The scalar-point multiplication and map-to-point operation are much more expensive than the other operations considered in the table, such as symmetric encryption/decryption, MAC generation/verification and hash function evaluation. The total number of modular exponentiations and map-to-point operations required in Yeh *et al.'s* scheme [16] is 10, while the number is reduced to six in the other schemes. Therefore, the overall performance of Yeh *et al.'s* scheme is not as good as those of the other schemes.

From the viewpoint of the computational burden on the sensor *SR*, our scheme is competitive with Choi *et al.*'s scheme [27] and Shi and Gong's scheme [21], since a MAC generation/verification is almost as fast as a hash function evaluation. According to Crypto++ benchmarks, HMACwith SHA-1 takes 11.9 cycles per byte, while SHA-1 takes 11.4 cycles per byte (see Table 4).

Another point we wish to make is that a hash function evaluation with a long input string may not be faster than a symmetric encryption with a relatively short plain text input, though the opposite is generally true for the same length of inputs. For example, the computation of the ciphertext *C_UR_* in our scheme is unlikely to be more expensive than the computations of the hash values *β, γ* and *δ*, which are defined in both Choi *et al.*'s scheme and Shi and Gong's scheme. In this sense, it is fair to say that our scheme is competitive also in terms of the overall computational cost.

As is obvious from the table, our scheme is the only one that provides user anonymity (regardless of whether it is proven or not). This explains how the other schemes could have been designed without using any form of encryption algorithm. Choi *et al.* [27] prove that their scheme achieves the AKE security, but only using a computer security approach. In contrast, we use a computational complexity approach in proving both the AKE security and the user anonymity property

## 4. Security Results

Let *P* denote the authentication and key exchange protocol of our scheme depicted in Figure 2. This section proves that the protocol *P* is AKE-secure and provides user anonymity (against any party other than the gateway *GW*); see Section 2 for the formal definitions of the AKE security and the user anonymity property

### 4.1. Proof of AKE Security

**Theorem 1.** *Our authentication and key exchange protocol P is AKE-secure in the random oracle model under the ECCDH assumption in 

 and the security of the MAC scheme* Δ.

**Proof.** Assume a ppt adversary 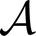 against the AKE security of the protocol *P*. We prove the theorem by making a series of modifications to the original experiment **ExpAKE**_0_, bounding the effect of each change in the experiment on the advantage of 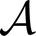 and ending up with an experiment where 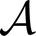 has no advantage (*i.e.*, 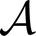 has a success probability of 1/2). Let SuccAKE*_i_* denote the event that 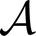 correctly guesses the random bit **b** selected by the **TestAKE** oracle in experiment **ExpAKE***_i_*. Let *t_i_* be the maximum time required to perform the experiment **ExpAKE***_i_* involving the adversary 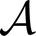.

Experiment **ExpAKE**_1_. In this first modified experiment, the simulator answers the queries to the *L* oracle as follows:

**Simulation of the *L* oracle**

For each query of *L* on a string *m*, the simulator first checks if an entry of the form (*m, l*) is in a list called LList, which is maintained to store input-output pairs of *L*. If it is, the simulator outputs *l* as the answer to the hash query. Otherwise, the simulator chooses a random ℓ-bit string *str*, answers the query with *str* and adds the entry (*m, str*) to LList.

This is the only difference between **ExpAKE**_1_ and **ExpAKE**_0_; the simulator answers all other oracle queries of 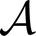 as in the original experiment **ExpAKE**_0_. Then, since **ExpAKE**_1_ is perfectly indistinguishable from **ExpAKE**_0_, it follows that:

**Claim 1.** Pr_*P*, 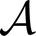
_[SuccAKE1] = Pr_*P*, 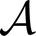
_[SuccAKE_0_].

Experiment **ExpAKE**_2_. In this experiment, we modify the computations of *X* and *A* as follows:

**The ExpAKE_2_ modification**

- The simulator chooses two random elements *Y,Y′* ∈ 

 and sets *X* = *Y′*.
- For every fresh instance, the simulator chooses a random $r\in {\mathbb{Z}}_{q}^{*}$ and sets *A* = *rY*. For other instances, the simulator computes *A* as in experiment **ExpAKE**_1_.

Due to the modification, the simulator does not know the master secret *x*. The simulator aborts the experiment if 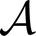 makes the CorruptLL(*GW*) query However, in this case, 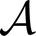 cannot gain any advantage, as no instance is considered fresh. In this experiment, the simulator simply sets each *k_UG_* to a random ℓ-bit string, since it does not know the ephemeral secret *a* and, thus, cannot compute the secret *K_Ug_*. This means that the success probability of 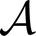 may be different between **ExpAKE**_1_ and **ExpAKE**_2_ if it asks an *L*(*T*_1_‖*A*‖*K_ug_*) query However, this difference is bounded by Claim 2.

**Claim 2.** $\left|{\mathrm{Pr}}_{P,A}\left[{\text{SuccAKE}}_{2}\right]-{\mathrm{Pr}}_{P,A}\left[{\text{SuccAKE}}_{1}\right]\right|\leq \overset{1}{\;}/\underset{qL}{\;}\cdot {\text{Adv}}_{\text{G}}^{\text{ECCDH}}\left(t_{2}\right)$, *where q_L_ is the number of queries made of the *L* oracle*.

**Proof.** We prove the claim via a reduction from the ECCDH problem, which is believed to be hard, to the problem of distinguishing two experiments **ExpAKE**_1_ and **ExpAKE**_2_. Assume that the success probability of 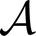 is non-negligibly different between **ExpAKE**_1_ and **ExpAKE**_2_. Then, we construct an algorithm 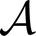
_ECCDH_ that solves the ECCDH problem in 

 with a non-negligible advantage. The objective of 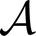
_ECCDH_ is to compute and output the value *W* = *uvP* ∈ 

 when given an ECCDH-problem instance (*U* = *uP, V* = *vP*) ∈ 

. 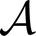
_ECCDH_ runs 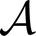 as a subroutine while simulating all of the oracles on its own.


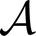
_ECCDH_ handles all of the oracle queries of 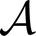 as specified in experiment **ExpAKE**_2_, but using *U* and *V* in place of X and *Y*. When 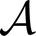 outputs its guess *b′*, 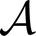
_ECCDH_ chooses an entry of the form (*T*_1_‖*A*‖ *K, l*) at random from LList and terminates outputting *K*/*r*. >From the simulation, it is not hard to see that 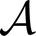
_ECCDH_ outputs the desired result *W* = *uvP* with probability at least 1/*_qL_* if 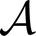 makes a *L*(*T*_1_‖*A*‖ *K_UG_*) query for some fresh user instance. This completes Claim 2.

Before proceeding further, we define the event Forge as follows:

Forge: The event that the adversary 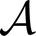 asks a Send query of the form $\text{Send}(\prod_{E}^{i},E^{\prime }∥\text{msg})$ for uncorrupted *E* and *E′*, such that *msg* contains a MAC forgery.

Experiment **ExpAKE**_3_. This experiment is different from **ExpAKE**_2_ in that it is aborted and the adversary 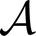 does not succeed if the event Forge occurs. Then, we have:

**Claim 3.** $\left|{\mathrm{Pr}}_{P,A}[{\text{SuccAKE}}_{3}]-{\mathrm{Pr}}_{P,A}[{\text{SuccAKE}}_{2}]\right|\leq q_{\text{send}}\cdot {\text{Adv}}_{\Delta }^{EU-\text{CMA}}(t_{3})$, *where q*_send_ *is the number of queries made for the oracle Send*.

**Proof.** Assume that the event Forge occurs with a non-negligible probability. Then, we construct an algorithm 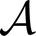
_forge_ who generates, with a non-negligible probability, a forgery against the MAC scheme Δ. The algorithm 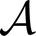
forge is given access to the Mack(·) and Verk(·) oracles. The objective of 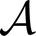
_forge_ is to produce a message/MAC pair (*m, δ*), such that: (1) Ver*_k_*(*m*, *δ*) = 1; and (2) *δ* has not been output by the oracle Mac*_k_*(·) on input *m*.

Let n*_k_* be the total number of MAC keys used in the sessions initiated via a Send query. Clearly, n*_k_* ≤ *q*_send_. 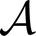
_forge_ begins by selecting a random *i* ∈ {1,…, n*_k_*}. Let *k_i_* denote the *i* – th key among all of the n*_k_* MAC keys and Sendi be any Send query that is expected to be answered and/or verified using *k_i_*. 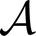
_forge_ runs 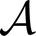 as a subroutine and answers the oracle queries of 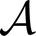 as in experiment **ExpAKE**_2_, except that: it answers all Sendi queries by accessing its Mac*_k_*(·) and Ver*_k_*(·) oracles. As a result, the *i* — th MAC key *k_i_* is not used during the simulation. If Forge occurs against an instance who holds *k_i_*, 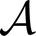
_forge_ halts and outputs the message/MAC pair generated by 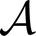 as its forgery. Otherwise, 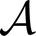
_forge_ terminates with a failure indication.

If the guess *i* is correct, then the simulation is perfect and 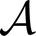
_forge_ achieves its goal. Namely, ${\text{Adv}}_{\Delta }^{EU-\text{CMA}}(A_{\text{forge}})=\mathrm{Pr}[\text{Forge}]/n_{k}$. Since n_k_ ≤ *q*_send_, we get $\mathrm{Pr}[\text{Forge}]\leq q_{\text{send}}\cdot {\text{Adv}}_{\Delta }^{EU-\text{CMA}}(A_{\text{forge}})$. Since 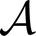
_forge_ runs in time at most *t*_3_, it follows, by definition, that ${\text{Adv}}_{\Delta }^{EU-\text{CMA}}(A_{\text{forge}})\leq {\text{Adv}}_{\Delta }^{EU-\text{CMA}}(t_{3})$. This completes the proof of Claim 3.

Experiment **ExpAKE**_4_. We next modify the way of answering queries of the *H* oracle as follows:

**Simulation of the *H* oracle**

For each *H* query on a string *m*, the simulator first checks if an entry of the form (*m*, *h*) is in a list called HList, which is maintained to store input-output pairs of *H*. If it is, *h* is the answer to the hash query. Otherwise, the simulator chooses a random *κ*-bit string *str*, answers the query with *str* and adds the entry (*m, str*) to HList.

Other oracle queries of 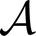 are handled as in experiment **ExpAKE**_3_. Since **ExpAKE**_4_ is perfectly indistinguishable from **ExpAKE**_3_, it is clear that:

**Claim 4.** Pr*_P, 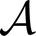
_*[SuccAKE_4_] = Pr*_P, 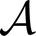
_*[SuccAKE_3_].

Experiment **ExpAKE**_5_. We finally modify the experiment so that, for each fresh instance of *SR*, the computation of *B* is done as follows:

**The ExpAKE_5_ modification**

The simulator selects a random $r^{\prime }\in {\mathbb{Z}}_{q}^{*}$ and computes *B* = *r′X*.

The simulator sets the session key *sk* to a random κ-bit string for each pair of fresh instances, as it cannot compute *K_SU_*. Accordingly, the success probability of 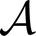 may be different between **ExpAKE**_4_ and **ExpAKE**_5_ if it asks an *H*(*A*‖*B*‖*K_SU_*) query. Claim 5 below bounds the difference:

**Claim 5.** $\left|{\mathrm{Pr}}_{P,A}[{\text{SuccAKE}}_{5}]-{\mathrm{Pr}}_{P,A}[{\text{SuccAKE}}_{4}]\right|\leq 1/q_{H}\cdot {\text{Adv}}_{G}^{\text{ECCDH}}(t_{5})$, *where qH is the number of queries made of the H oracle*.

**Proof.** Suppose that the difference in the advantage of 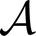 between SuccAKE_4_ and SuccAKE_5_ is non-negligible. Then, from 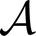, we construct an algorithm 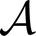
_ECCDH_ that solves the ECCDH problem in 

 with a non-negligible advantage. The objective of 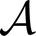
_ECCDH_ is to compute and output the value *W* = *uvP* ∈ 

 when given an ECCDH-problem instance (*U* = *uP, V* = *vP*) ∈ 

.


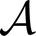
_ECCDH_ runs 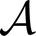 as a subroutine while answering all of the oracles queries by itself. 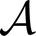
_ECCDH_ handles the queries of 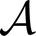 as specified in the **ExpAKE**_5_ experiment, but using *U* and *V* in place of *X* and *Y*. When 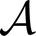 terminates and outputs its guess *b′*, 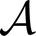
_ECCDH_ selects an entry of the form (*A*‖*B*‖*K, h*) at random from HList and outputs *K/rr′*. If 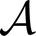 makes a *H*(*A*‖*B*‖*K_SU_*) query, 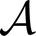
_ECCDH_ outputs the desired result *W* = *uvP* with probability at least 1/*q_H_*. This completes Claim 5.

In experiment **ExpAKE**_5_, the adversary 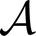 obtains no information on the random bit *b* selected by the TestAKE oracle, since the session keys of all fresh instances are selected uniformly at random from 

. Therefore, it follows that Pr*_P_*, 

[SuccAKE_5_] = 1/2. This result combined with Claims 1–5 completes the proof of Theorem 1.

### 4.2. Proof of User Anonymity

**Theorem 2.** *The authentication and key exchange protocol P provides user anonymity in the random oracle model under the ECCDH assumption in 

 and the security of the symmetric encryption scheme* Γ.

**Proof.** Assume a PPT adversary 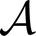 against the user anonymity property of the protocol P. As in the proof of Theorem 1, we make a series of modifications to the original experiment **ExpID**_0_, bounding the difference in the success probability of 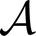 between two consecutive experiments and then ending up with an experiment where 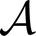 has a success probability of 1/2 (*i.e.*, 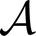 has no advantage). We use SuccID*_i_* to denote the event that A correctly guesses the random bit b selected by the TestID oracle in experiment **ExpID**_i_. Let *t_i_* be the maximum time required to perform the experiment **ExpID**_i_ involving the adversary 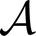.

Experiment **ExpID**_1_. This experiment is different from **ExpID**_0_ in that the random oracle *L* is simulated as follows:

**Simulation of the *L* oracle**

For each query to *L* on a string *m*, the simulator first checks if an entry of the form (*m, l*) is in a list called LList, which is maintained to store input-output pairs of *L*. If it is, the simulator outputs *l* as the answer to the hash query. Otherwise, the simulator chooses a random ℓ-bit string *str*, answers the query with *str* and adds the entry (*m, str*) to LList.

Other oracle queries of 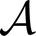 are answered as in the original experiment **ExpID**_0_. Then, since *L* is a random oracle, **ExpID**_1_ is perfectly indistinguishable from **ExpID**_0_, and Claim 6 immediately follows.

**Claim 6.** Pr*_P_*, 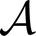
[SuccID_1_] = Pr_P_, 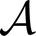
[SuccID_0_].

Experiment **ExpID**_2_. Here, we modify the experiment so that A is computed as follows:

**The ExpID_2_ modification**

- The simulator chooses a random exponent $y\in {\mathbb{Z}}_{q}^{*}$ and computes *Y* = *y P*.
- For each instance of users, the simulator chooses a random $r\in {\mathbb{Z}}_{q}^{*}$ and sets *A* = *rY*.

As a result of the modification, each *K_Ug_* is set to *xyrP* for some random $r\in {\mathbb{Z}}_{q}^{*}$. Since the view of 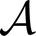 is identical between **ExpID**_2_ and **ExpID**_1_, it follows that:

**Claim 7.** Pr*_P_*, 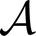
[SuccID_2_] = Pr*_P_*,_
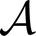
_[SuccID_1_].

Experiment **ExpID**_3_. In this experiment, we modify the computations of *X* and *A* as follows:

**The ExpID_3_ modification**

- The simulator chooses two random elements *Y,Y′*∈ 

 and sets *X* = *Y′*.
- For instances of every clean user, the simulator chooses a random $r\in {\mathbb{Z}}_{q}^{*}$ and sets *A* = *rY*. For other instances, the simulator computes *A* as in experiment **ExpID**_2_.

As a result, the simulator does not know the master secret *x*. The simulator aborts the experiment if 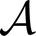 makes the CorruptLL(GW) query However, in this case, 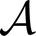 cannot gain any advantage, as no user is considered clean (see Definition 3). In this experiment, the simulator simply sets each *k_UG_* to a random ℓ-bit string, since it does not know the ephemeral secret *a* and, thus, cannot compute the secret *K_ug_*. This means that the success probability of 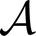 may be different between **ExpID**_2_ and **ExpID**_3_ if it asks an *L*(*T*_1_‖*A*‖*K_Ug_*) query However, this difference is bounded by Claim 8.

**Claim 8.** $\left|{\mathrm{Pr}}_{P,A}[{\text{SuccID}}_{3}]-{\mathrm{Pr}}_{P,A}[{\text{SuccID}}_{2}]\right|\leq 1/q_{L}\cdot {\text{Adv}}_{\text{G}}^{\text{ECCDH}}(t_{3})$ *where q_L_ is the number of queries made to the *L* oracle*.

**Proof.** We prove the claim via a reduction from the ECCDH problem, which is believed to be hard, to the problem of distinguishing two experiments **ExpID**_2_ and **ExpID**_3_. Assume that the success probability of 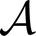 is non-negligibly different between **ExpID**_2_ and **ExpID**_3_. Then, we construct an algorithm 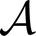
_ECCDH_ that solves the ECCDH problem in 

 with a non-negligible advantage. The objective of 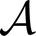
_ECCDH_ is to compute and output the value *W* = *uvP* ∈ 

 when given an ECCDH-problem instance (*U* = *uP, V* = *vP*) ∈ 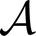
_AECCDH_ runs 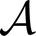 as a subroutine while simulating all of the oracles on its own.


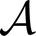
_ECCDH_ handles all of the oracle queries of 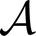 as specified in experiment **ExpID**_3_, but using *U* and *V* in place of *X* and *Y*. When 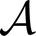 outputs its guess *b′*, 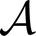
_ECCDH_ chooses an entry of the form (*T*_1_‖*A*‖*K, l*; at random from LList and terminates outputting *K/r*. >From the simulation, it is clear that 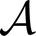
_ECCDH_ outputs the desired result *W* = *uvP* with a probability of at least 1/*qL* if *A* makes a L(*T*_1_‖*A*‖*K_UG_*) query for some clean *UR* ∈ 
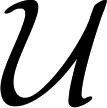
ℛ 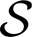. This completes Claim 8.

Experiment **ExpID**_4_. We finally modify the experiment so that, for each clean user *UR* ∈ 
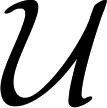
ℛ 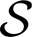, a random identity *ID′_UR_* drawn from the identity space is used in place of the true identity *ID_UR_* in generating *C_UR_*.

**Claim 9.** $\left|{\mathrm{Pr}}_{P,A}\left[{\text{SuccID}}_{4}\right]-{\mathrm{Pr}}_{P,A}\left[{\text{SuccID}}_{3}\right]\right|\leq {\text{Adv}}_{\Gamma }^{\text{IND}-\text{EAV}}\left(t_{4}\right)$.

**Proof.** We prove the claim by constructing an eavesdropper 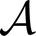
_eav_ who attacks the indistinguishability of Γ with advantage equal to |Pr_*P*, 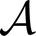
_[SuccID4] − PrP, 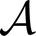
[SuccID3]|.


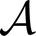
_eav_ begins by choosing a random bit *b* ∈ {0,1}. Then, 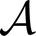
_eav_ invokes the adversary 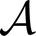 and answers all of the oracle queries of 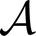 as in experiment ExpID3, except that, for each clean user *UR* ∈ 
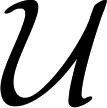
ℛ 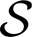, it generates *C_UR_* by accessing its own encryption oracle as follows:

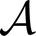
_eav_ outputs (*SID_UR_*‖*ID_UR_*‖*ID_SR_, SID_UR_*‖*ID′UR*‖*ID_SR_*) as its plain text pair in the indistinguishability experiment ${\text{Exp}}_{\Gamma }^{\text{IND}-\text{EAV}}$. Let *c* be the ciphertext received in return for the plain text pair. 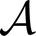
eav sets Cur equal to the ciphertext *c*.

That is, 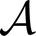
_eav_ sets *C_UR_* to the encryption of either *SID_UR_*‖*ID_UR_*‖*ID_SR_* or *SID_UR_*‖*ID_UR_*‖*ID_SR_*. Now, when 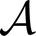 terminates and outputs its guess *b′*, 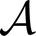
eav outputs one if *b* = *b′*, and zero otherwise. Then, it is clear that:

- The probability that 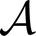
_eav_ outputs one when the first plain texts are encrypted in the experiment ${\text{Exp}}_{\Gamma }^{\text{IND}-\text{EAV}}$ is equal to the probability that 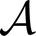 succeeds in the experiment **ExpID**_3_.
- The probability that 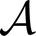
_eav_ outputs one when the second plain texts are encrypted in the experiment ${\text{Exp}}_{\Gamma }^{\text{IND}-\text{EAV}}$ is equal to the probability that 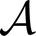 succeeds in the experiment **ExpID**_4_.

That is, ${\text{Adv}}_{\Gamma }^{\text{IND}-\text{EAV}}\left(A_{\text{eav}}\right)=\left|{\mathrm{Pr}}_{P,A}\left[{\text{SuccID}}_{4}\right]-{\mathrm{Pr}}_{P,A}\left[{\text{SuccID}}_{3}\right]\right|$. Note that in the simulation, 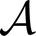
_eav_ eavesdrops at most *q*_send_ encryptions, which is polynomial in the security parameter ℓ. This completes the proof of Claim 9.

In the experiment **ExpID**_4_, the adversary 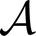 cannot gain any information on the random bit *b* selected by the TestID oracle, because the identities of all clean users are chosen uniformly at random from the identity space. It, therefore, follows that Pr_P, 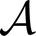
_[SuccID_4_] = 1/2. This result combined with Claims 6–9 yields the statement of Theorem 2.

## 5. Concluding Remarks

We have extended the widely-accepted security model of Bellare, Pointcheval and Rogaway [10] to formally capture the security requirements for SUA-WSN schemes—smart-card-based user authentication schemes for wireless sensor networks. Our extended model provides formal definitions of the AKE security and the user anonymity property, while capturing the notion of two-factor security. We have also proposed a new SUA-WSN scheme and proved that it achieves user anonymity, as well as the AKE security in the extended model. To the best of our knowledge, our scheme is the first SUA-WSN scheme that is proven secure in a widely-accepted model.

We believe that our result lays a solid foundation for designing provably-secure two-factor authentication schemes for mobile roaming services, where user anonymity, as well as authenticated key exchange are also of critical security importance; see, e.g., the recent work of He *et al.* [33,34]. A concrete design of such a provably-secure roaming authentication scheme would be interesting future work. We also leave it as future work to present a formal treatment of security properties for three-factor authentication schemes [35].

## Figures and Tables

**Table 1.** A summary of security results for existing SUA-WSN (smart-card-based user authentication scheme for wireless sensor networks) schemes.

**Scheme**	**Security Justification**	**Major Weaknesses**
Das [12]	Heuristic arguments	No key-exchange functionality
He *et al.* (2010) [13]	Heuristic arguments	No key-exchange functionality
Khan and Alghathbar [14]	Heuristic arguments	No key-exchange functionality
Chen and Shih [15]	Computational complexity approach (only for entity authentication)	No key-exchange functionality
Yeh *et al.* [16]	Heuristic arguments	Failures of mutual authentication and forward secrecy [30]
Kumar *et al.* (2011) [2]	Computer security approach	Vulnerability to a node capture attack [24]
Kumar *et al.* (2012) [17]	Computer security approach	Failures of authenticated key exchange, user anonymity and two-factor security [3,23]
Yoo *et al.* [18]	Computer security approach	Vulnerability to a man-in-the-middle attack [22]
Vaidya *et al.* [19]	Computer security approach	Failure of user authentication [25]
Xue *et al.* [20]	Heuristic arguments	Vulnerability to a privileged insider attack [26]
Shi and Gong [21]	Heuristic arguments	Failures of authenticated key exchange and two-factor security [27]
Kumar *et al.* (2013) [22]	Heuristic arguments	
He *et al.* (2013) [23]	Computer security approach	
Chi *et al.* [24]	Heuristic arguments	
Kim *et al.* [25]	Heuristic arguments	
Khan and Kumari [3]	Heuristic arguments	
Jiang *et al.* [26]	Heuristic arguments	
Choi *et al.* [27]	Computer security approach	No provision of user anonymity

**Table 2.** Basic notation.

**Symbol**	**Description**
*UR*	User
*SR*	Sensor
*GW*	Gateway
*ID_UR_,ID_SR_,ID_GW_*	Identities of *UR, SR* and *GW*
*pw_UR_*	Password of *U*
*sk*	Session key
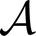	Probabilistic polynomial-time adversary
*L*(·),*H*(·),*F*(·)	Cryptographic hash functions
Enc*_k_*(·)/Dec*_k_*(·)	Symmetric encryption/decryption under key *k*
MAC	Message authentication code
Mac*_k_*(·)/Ver*_k_*(·)	MAC generation/verification under key *k*
⊕	Bitwise exclusive-or (XOR) operation
‖	String concatenation operation
{0,1}*^n^*	Bit strings of length *n*

**Table 3.** A comparison of elliptic curve cryptography (ECC)-based SUA-WSN schemes. AKE, authenticated key exchange.

**Scheme**	**Computation**		**Security**	
	
	**SR**	**UR+SR+GW**	**AKE**	**Anonymity**
Our scheme	2*M* +2*A*+1*H*	6*M* +3*E* +6*A*+6*H*	Proven	Proven

Choi *et al.* [27]	2*M* +5*H*	6*M* +18*H*	Proven using a computer security approach	No

Shi and Gong [21]	2*M* +4*H*	6*M* +15*H*	Broken [27]	No

Yeh *et al.* [16]	2*M* +1*P* + 2*H*	8*M* +2*P* +9*H*	Broken [30]	No

*M*: scalar-point multiplication; *P*: map-to-point operation; *E*: symmetric encryption/decryption; *A*: MAC generation/verification; *H*: hash function evaluation.

**Table 4.** A result of Crypto++ benchmarks for HMAC, SHA-1 and AES.

**Algorithm**	**HMAC (SHA-1)**	**SHA-1**	**AES/CTR**	**AES/CBC**	**AES/OFB**	**AES/ECB**
Cycles Per Byte	11.9	11.4	12.6	16.0	16.9	16.0
